# Conservative Dentistry

**Published:** 1871-06

**Authors:** George H. Cushing


### ARTICLE II.

### *Conservative Dentistry.*

By GEORGE H. CUSHING, D.D.S.

Read before the Chicago Dental Society.

The subject assigned to me for this evening, is one, the importance of which I think is not properly appreciated, and in attempting its treatment, I am met at the outset with the inquiry which I know is made inwardly by many minds if not outwardly by many lips: "What is conservative dentistry?"

I should not perhaps have thought it necessary to attempt any elaborate definition of the term, if I had not heard two gentlemen of the profession, among the most intelligent and advanced of my acquaintance, making the inquiry as to the meaning of the term which constitutes the heading of this paper; so, though the matter seemed so clear to my own mind, I have upon reflection concluded that it is very probable that the term may convey little significance to many minds, and, therefore, demands that it should be as briefly defined as is consistent with a fair understanding of the subject.

Conservative is defined by Webster as "having power to preserve in a safe or entire state, or from loss, waste or injury—preservative." It comes from the Latin "con," and "servare," "to keep, to guard." The term has been greatly in vogue for several years past, as applied to general surgery, though it is doubtful if any uniformity of definition prevails among medical men.

Prof. Rae, of Rush Medical College, says: "Conservative surgery is conventional, meaning, as I understand it, preservative surgery, and is generally applied to the place between extremes. It is usually applied as antipode to heroic surgery."

Professor Gross in his work, in the chapter on "Excision of Bones and Joints," says: "Excision differs from amputation in this, that while in the latter the bone is removed along with the soft parts which surround it, in the former the bone alone is cut away, the integuments, muscles and other tissues being retained, in order that they may contribute to the future usefulness of the limb, or, in other and more comprehensive language, while in the one case all the structures are destroyed, in the other as many of them as possible are preserved. Hence this department of surgery has very appropriately been denominated 'conservative surgery,' and it is gratifying to know that it constitutes one of the leading characteristics of the healing art of the present day." He thus seems to confine the term, in its application, to that especial division of surgery.

Again, Dr. Davis, of New York, has published a work entitled "Conservative or Mechanical Surgery, as exhibited in remedying some of the mechanical causes that operate injuriously, both in health and disease." Thus, there would seem to be no authoritative definition of the term among surgeons, yet I believe there is a broader and deeper significance to the term than any of the definitions above given would seem to imply, and one which is generally felt among surgeons, although perhaps difficult to define. I think it clearly has reference to those advanced methods of practice, which have of later years become so general in any and all departments of surgery, which tend to lessen the frequency or operations involving great loss of structure, either by substituting less formidable ones, or by anticipating disease, so that in many cases no operation at all is necessary.

The surgeon following this higher order of practice thus becomes the conserver—preserver—his province is, "to keep" —"to guard"—not only the life and health of his patient, but also the integrity of his body to the very fullest extent.

If, then, there is this important application of the term conservative in relation to general surgery, how much more emphatically should it apply, as in reference to the specialty of dentistry.

It is true that the dentist has not to operate on organs or parts of the body involving, as a rule, such important relations to the vital functions, or locomotion, or to general usefulness, as the general surgeon has ; nor does his operations often threaten danger to life or general health, but his field is a very important one, and upon his successful operations, or otherwise, often depends to a great extent, not only the comfort, but the health of his patient, and his skill should be as conscientiously applied as though he were operating on more vital organs. He is constantly called upon to decide between the immediate loss of organs of the economy, or the effort to preserve them. In his case it is utter loss of those organs on the one hand, as against the attempt to preserve them on the other, while there is not, as is often the case with operations in general surgery, the danger of fatal consequences to be weighed in forming a decision.

There are those in the profession who hold that there is a moral obligation binding upon all men, not to mutilate the human body in the slightest degree beyond what is actually demanded as a "dernier resort," to remove otherwise incurable disease, or in case of extreme suffering, or the removal of obnoxious deformities. Such men will tell you "it is a sin to extract a tooth." Without taking the extreme position which this class occupy, I will say that there is more truth in their assumptions than the profession generally are willing to acknowledge. The province of the dentist is essentially that of a "conserver"—a "preserver." He is in duty bound "to keep" and "to guard" that portion of the of the human body which it is his province to treat, as much as for the general surgeon to save to the uttermost that which lies within the province of his art to treat.

Let us then inquire as to the present status of the profession as regards this sort of practice—how we stand as in relation to the past, and what can be forecast regarding the future—touching briefly some of the prominent points in relation to the subject which seem to demand especial consideration.

The time was, and not more than twenty years ago, when the prevailing practice was to extract most aching teeth, except perhaps the six anterior teeth in either jaw,—or, if such teeth were not extracted, the majority of honest practitioners gave little encouragement to the patient for any prolonged usefulness of a tooth, the pulp of which had to be devitalized before filling. Dating perhaps from that time, advancement began to be apparent in the methods of treatment of teeth with exposed pulps, and greater thoroughness in the operations of removing the devitalized pulps and filling of the pulp chamber and canals, brought with it greater encouragement as to this method, until a few years later the thorough and conscientious practitioner could honestly advise his patients to have this operation performed, as promising reasonably of success. This was eminently an advance in "conservative practice," and so marked that I doubt not all of you here present can corroborate the statement, that while, even ten years ago, the usual remark of the patient when coming to us with an aching tooth was: "Doctor, I have an aching tooth, I want you to extract it,"—that to-day the large majority say instead, "Doctor, I have a bad tooth, I wish you would see if you can do anything to save it." And while two years ago it was very difficult to persuade some patients—and very many, too—to consent to the attempt to save the tooth by devitalization of the pulp, to-day it is sometimes difficult to persuade people to have a tooth extracted, even when there is obviously no other course proper to be pursued.

This is evidence of remarkable advancement, for when you find the people educated to the point of demanding such operations, you may be sure that the operations have met with favor, through the established evidence of their reasonable success.

Recognizing the demands of this conservative principle, earnest men in the profession, even as long ago as twenty-five years, or thereabouts, turned their attention toward devising some method of treatment of exposed pulps, which should render it possible to fill such teeth, and retain at the same time their vitality. Various methods of capping exposed pulps were pursued, and some with occasional seeming successes, but after years of experiment and experience, the profession came to decide generally that the capping of exposed pulps was not a reliable practice, and it was in the main abandoned, and devitalization and extirpation held almost undisputed possession of the field, until the introduction to the notice of the profession of the method of capping with oxy-chloride zinc. This method has seemed to prove thus far, more successful than any previously tried, and although it has not yet been tested by sufficient length of time to justify us in pronouncing fully upon its merits, it certainly can be said to give great promise for the future, and through its aid we may reasonably hope to arrive at the highest point to be attained in "conservative practice."

These are the most striking features which present themselves in a review of this subject, but there are others almost as important, and in connection with which there are some points which especially demand our consideration.

The introduction of cohesive foil and gold, inaugurated a marked era in dental art, rendering possible the performance of very many operations which otherwise, or with soft foil, could never have been performed. In this way the crowns of teeth can be restored, and the organs rendered reasonably permanent, while otherwise many such teeth could not be successively treated, and would early fall a prey to the forceps, and others could only be treated with partial success by the use of amalgam, that always uncertain material, even if there were no other objections to its general use.

The class of operations here referred to have frequently been derisively termed "*fancy operations.*"

They are fancy operations in just the sense that the "excisions of joints" are fancy operations, as compared with amputation of the entire limb, of which Professor Gross—as before quoted—says, "it is most gratifying to know that it constitutes one of the leading characteristics of the healing art of the present day." I am glad to be able to say, that the same expression may be used as applied to our specialty, for such operations are eminently conservative, and are daily coming to be better understood and more justly appreciated.

The improved methods of manipulating cohesive foil, together with the superior character of instruments of late years introduced, which have come to us the result of earnest thought and experiment, tested by experience from leading men in the profession, leave no excuse for most men to use any other material than gold for filling in a large majority of cases. And yet, notwithstanding the great advance which it has been affirmed has been made toward true conservative practice, we find to-day, very often, in large cities particularly, a class of operators, and first-rate operators as well, who use amalgam to an alarming extent. Men who command first-rate fees too.

You will often hear such men say, "I frequently hear of Dr. A. or B. charging forty, fifty or sixty dollars for an operation, but I never could bring my conscience to the point of making such charges." To those acquainted with the operations of such men, the reason is very obvious—they never perform the operations which entitle a man to such a fee. In such cases they invariably use amalgam, when gold could be used to much greater advantage to the patient, and where, did they use the gold, they would find that their consciences would not only allow them to charge such fees, but that they could not conscientiously perform the operations for less.

It is just here that I think a large part of the profession fails to appreciate the demands of the conservative principle; neglecting to do their duty by their patients, either through fear to demand a just recompense for their skill and labor, or from disinclination to make operations demanding so great an outlay of time and nervous force. There are many practitioners who follow this course through ignorance, and sin blindly; but for such there can be no excuse offered, because if they attend the Societies faithfully, take and read all the journals studiously, and inform themselves as fully as they can, it will be made clear to them that such operations as those just referred to can be made with gold successfully, and of a much more lasting character than with any other material and they should learn to do these things, nor rest satisfied until they can perform successfully the operations which so many around them have long been and are still constantly performing.

I wish particularly to call attention to this point, and not so much that of the younger members of the profession, as of the older—for to the latter, as a general rule, the temptation comes with much more force, to shrink the responsibility and fatigue of such operations, than to the younger members. The older members generally feel perhaps that their reputations are established, while the younger have theirs still to build up.

Again, the temptation comes to most of us almost daily to extract teeth which we feel assured may be preserved by careful pains-taking treatment.

We dread to undertake operations which we know will be tedious and discouraging, both to the patient and to ourselves ; and so many times we try to convince ourselves against our real convictions, that the best that we can do for our patients is to advise extraction. This we surely have no right to do, for we are not thus doing our highest duty—if there is anything higher than simple duty—and that duty is to be found in simply living up to the conservative principle.

It may be safely affirmed, as a general rule, that where there is reasonable hope of ultimate success, the attempt should always be made to save a tooth, and it should be restored as fully as possible to its original usefulness. Anything short of this for our aim is not truly conservative.

These are the main points to which I desire to call your attention, but it must not be forgotten that prevention is better than cure, and that the highest expression of conservative practice will be reached only when we can successfully address ourselves to prevention rather than cure.

What I have said imperfectly upon this subject has been with a view to enunciate a great principle, and is not to be understood as condemning *in toto* certain practices, as for instance, the use of amalgam, which under certain circumstances is unquestionably invaluable, or with reference to extraction, for clearly that is frequently demanded ; but it is affirmed that according to our light our endeavor should always be conservative.

The mistake must not be made in the application of the term conservative, in its sense of being opposed to change or innovation—which is one of its definitions—for conservative practice in surgery or any of its branches depends for its very life upon change and innovation—it is essentially progressive.

The endeavor has been made to place this subject before you in a manner that might be comprehended, and with the hope that the importance of it might be impressed upon your minds. In that hope I shall leave the subject, summing up briefly the definition of conservative dentistry as the conscientious application of the highest attainments in our science and art, tending always to the conservative—the preservation of the teeth in a safe or entire state, keeping and guarding them from greater loss of structure than is absolutely essential they should suffer in the process of restoring and saving them.

The advance which has been made in this direction during the past ten years, gives us great encouragement to hope for the future, that conservative dentistry will ultimately attain to a near approach to perfection, and rank deservedly among the highest specialties of surgery.

To this end we should not forget that every individual owes it to his profession, himself and the public, to advance by every means in his power, the science and art of his chosen calling, that that day may the sooner arrive, when conservative dentistry shall be crowned with the well-deserved praise of carrying healing and beneficence on its wings.

---

